# Regioselective Localization and Tracking of Biomolecules on Single Gold Nanoparticles

**DOI:** 10.1002/advs.201500232

**Published:** 2015-09-28

**Authors:** Bharath Bangalore Rajeeva, Derek S. Hernandez, Mingsong Wang, Evan Perillo, Linhan Lin, Leonardo Scarabelli, Bharadwaj Pingali, Luis M. Liz‐Marzán, Andrew K. Dunn, Jason B. Shear, Yuebing Zheng

**Affiliations:** ^1^ Department of Mechanical EngineeringMaterials Science and Engineering ProgramTexas Materials InstituteThe University of Texas at AustinAustinTX78712USA; ^2^ Department of ChemistryThe University of Texas at AustinAustinTX78712USA; ^3^ Department of Biomedical EngineeringThe University of Texas at AustinAustinTX78712USA; ^4^ Bionanoplasmonics LaboratoryCIC biomaGUNEPaseo de Miramón 18220009Donostia–San SebastiánSpain; ^5^ IkerbasqueBasque Foundation for Science48013BilbaoSpain

**Keywords:** biomolecules, gold nanotriangle, multiphoton plasmonic lithography, single‐particle spectroscopy

## Abstract

Selective localization of biomolecules at the hot spots of a plasmonic nanoparticle is an attractive strategy to exploit the light–matter interaction due to the high field concentration. Current approaches for hot spot targeting are time‐consuming and involve prior knowledge of the hot spots. Multiphoton plasmonic lithography is employed to rapidly immobilize bovine serum albumin (BSA) hydrogel at the hot spot tips of a single gold nanotriangle (AuNT). Regioselectivity and quantity control by manipulating the polarization and intensity of the incident laser are also established. Single AuNTs are tracked using dark‐field scattering spectroscopy and scanning electron microscopy to characterize the regioselective process. Fluorescence lifetime measurements further confirm BSA immobilization on the AuNTs. Here, the AuNT‐BSA hydrogel complexes, in conjunction with single‐particle optical monitoring, can act as a framework for understanding light–molecule interactions at the subnanoparticle level and has potential applications in biophotonics, nanomedicine, and life sciences.

## 1 Introduction

The interaction of incident electromagnetic radiation with metallic nanoparticles results in collective electron oscillations, known as localized surface plasmon resonances (LSPRs). A consequence of the interaction is the ability to concentrate light fields with orders of magnitude enhancement at visible and near infrared (NIR) wavelengths to the subwavelength scale. Such enhancements find profound applications in improving the efficiencies of several phenomena such as fluorescence,^1^, ^2^, ^3^ surface‐enhanced Raman spectroscopy (SERS),^4^, ^5^, ^6^, ^7^, ^8^ surface‐enhanced infrared absorption,^9^, ^10^, ^11^ single‐molecule detection,^12^, ^13^ nonlinear optical effects,^14^, ^15^, ^16^ and multiphoton polymerization.^17^, ^18^ Exploiting this highly confined optical field is crucial, but simultaneously it is also challenging to position the desired molecules or particles accurately at these locations. Different approaches have been employed to achieve selective positioning of the molecules over defined hot spots, such as (i) incorporation of a two‐step electron beam exposure and squeegee process,^19^ where the first exposure step creates the plasmonic nanostructure and the second step selectively generates openings at hot spots,^20^ (ii) integrating a microfluidic channel with appropriate flow control,^21^ (iii) use of atomic force microscopy to manipulate the position of nanoparticles,^22^ (iv) exploiting material selective surface chemistry for attachment,^23^, ^24^ and (v) using multiphoton plasmonic lithography (MPPL) to induce selective polymerization at hot spots due to the enhanced electromagnetic field of the nanostructures.^17^, ^25^, ^26^, ^27^ Among the above strategies, techniques (i)–(iv) are time‐intensive, prone to inaccuracies, and require prior knowledge of the precise hot spot location. In contrast, MPPL utilizes the inherent field enhancement of nanostructures to localize the desired molecules into the hot spots, making this approach fast and reliable.

MPPL or metal‐enhanced multiphoton absorption has been primarily used in conjunction with commercial photoresists such as SU‐8 and TSMR V‐90 due to their potential applications in achieving highly resolved features.^17^, ^25^, ^28^, ^29^, ^30^ MPPL has also been studied to visualize plasmon modes in nanostructures.^31^, ^32^ In recent years, understanding molecule–plasmon interactions has gathered attention for applications such as plasmonic switches^33^, ^34^, ^35^ and nanoplasmonic rulers.^36^, ^37^ Advancing this application toward immobilizing biomaterials such as proteins would enable the exploration of biological phenomena at the nanoscale, where numerous cell–matrix interactions occur. Numerous proteins have been cross‐linked using multiphoton lithography (MPL), and many of these proteins were shown to retain their bioactivity; however, the resolution of current systems, ≈500 nm, is much larger than individual cell surface receptor interactions.^38^, ^39^, ^40^, ^41^ One particular protein/photosensitizer platform that has been regularly employed to fabricate densely cross‐linked, 3D protein hydrogels is bovine serum albumin (BSA) in combination with Rose Bengal (RB).^42^ Recently, Galloway et al. used light‐assisted molecular immobilization (LAMI) to immobilize BSA within gold dimers to selectively direct the attachment of single Au nanoparticles.^43^ While LAMI requires longer fabrication times to dehydrate proteins, BSA hydrogels can be deposited in a single step without considerable waiting time, using MPPL. Also, other techniques such as stimulated emission depletion lithography do not have control over the position and are limited to ≈100 nm resolution.^44^

Further, it is challenging to characterize changes occurring on single nanoparticles at the nanoscale. Conventional spectroscopic techniques provide limited information due to particle size heterogeneity and the relatively low concentration of molecules immobilized over the hot spots.^30^ In contrast, single‐nanoparticle dark‐field scattering spectroscopy records the spectrum of single nanoparticles originating from LSPRs.^45^, ^46^ Thus, it is extremely sensitive to morphology and the refractive index variations at the particle surfaces, aiding in tracking changes over single nanoparticles.^47^, ^48^ In our study, dark‐field scattering microscopy provides valuable information regarding the immobilization of proteins on single nanoparticles.

In the present work, we utilized MPPL using a focused titanium:sapphire femtosecond laser to selectively and rapidly (≈20 s to completely expose an area of (40 × 40) μm^2^) localize cross‐linked BSA hydrogels at the tips of gold nanotriangles (AuNTs). The tips of the AuNTs are the sites of maximum electromagnetic field intensity enhancement, a necessary element to induce cross‐linking. The AuNTs are immobilized on silanized glass coverslips with ample interspacing to ensure that the single particles can be distinguished in dark‐field scattering microscopy. The shape of the examined AuNTs was confirmed via correlated scanning electron microscopy (SEM). We demonstrate site selectivity and control over the extent of hydrogel fabrication on AuNT tips, processes that depend on the polarization and intensity of the incident laser, respectively. In addition, strong intensity dependency, short fabrication times, and extensive rinsing greatly reduced nonspecific protein adsorption on the substrate. Protein immobilization was tracked with single‐particle dark‐field scattering microscopy by recording the scattering spectra of an AuNT before and after the fabrication. The magnitude of the peak shift was found to be in positive correlation with the volume of protein present at the hot spots. Correlated fluorescence lifetime imaging microscopy (FLIM) was also performed to confirm protein immobilization.

## 2 Results and Discussion

**Figure 1:**
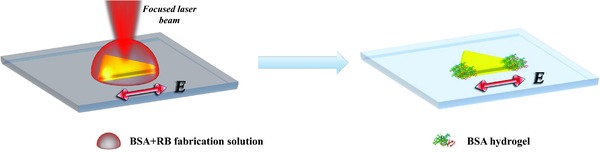
Schematic representation of the selective localization of BSA hydrogel molecules at the tip of a single AuNT. The protein is cross‐linked close to the tip via MPPL, and not elsewhere.

The immobilization of BSA hydrogel was performed over well‐dispersed AuNTs on a silanized glass coverslip. Colloidal solutions of AuNTs with a uniform edge length distribution of 59 ± 4 nm (see Figure S1, Supporting Information, for the absorbance spectra) were prepared using a previously reported procedure^49^ (see the Experimental Section for details) and casted on the coverslip. The AuNT solution was centrifuged and redispersed in water twice to minimize the concentration of surfactant, hexadecyltrimethylammonium chloride (CTAC). BSA hydrogels were fabricated using MPL in an aqueous environment. The defining aspect of MPL is that photochemical cross‐linking only occurs near the focal point of the incident laser beam, where laser intensity is high enough for photoreactants to absorb two or more photons near simultaneously, a phenomenon known as nonlinear absorption. RB, the photosensitizer used in these studies, undergoes facile intersystem crossing, and is believed to promote intermolecular protein crosslinking through singlet‐oxygen and/or direct hydrogen‐transfer mechanism.^50^ Hydrogel fabrication performance is mainly dependent on the concentration of components in the fabrication solution and the intensity of the incident laser, with a higher concentration of RB likely resulting in formation of a greater number of protein cross‐links. The intensity dependence is crucial in the present work, since the two‐photon process requires a sufficiently high photon flux for nonlinear excitation to occur. This intensity dependence is exploited in case of MPPL wherein the field enhancement of the AuNT amplifies the energy at the tips, resulting in an enhanced energy density within these regions that exceeds the threshold exposure (**Figure** **1**).^17^

**Figure 2:**
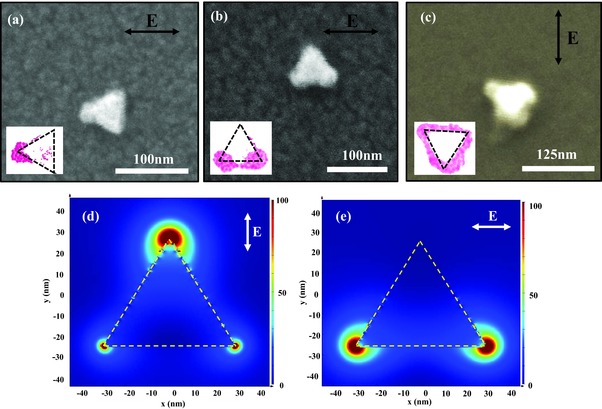
SEM images showing the regioselective localization of BSA hydrogel on single AuNTs under various incident light polarization and intensity at a,b) incident laser power of 1 mW and c) incident laser power of 2 mW. Inset in (a–c) shows the BSA hydrogel positions isolated via image processing. The direction of the electric field vector of the incident laser is denoted at the top‐right of each figure. d,e) Simulated field intensity distributions over the surface of AuNT. The intensity enhancement (|*E*|/|*E*_0_|) is depicted on the color scale, indicating higher intensity at one or two tips, depending on the polarization.

**Figure** **2** shows high‐magnification SEM images of the successful fabrication of AuNT‐BSA hydrogel hybrids using MPPL, and the corresponding field intensity distributions obtained by finite‐difference time domain (FDTD) simulations using a commercial package by Lumerical Inc. In Figure 2a,b, fabrication was performed with an average laser power of 1 mW, whereas in Figure 2c, the laser power was 2 mW. All powers were measured at the back aperture of the objective. The low milliwatt powers at the objective back aperture yield peak laser intensities within the high numerical aperture focal volumes of ≈10^10^–10^12^ W cm^−2^. Such powers are considerably lower than the typical requirements for direct laser fabrication, which require a minimum of ≈14 mW with our scan parameters. Incident laser powers above 2 mW resulted in complete coverage of the AuNT with the BSA hydrogel. In order to clearly isolate the locations of BSA hydrogels, the high‐magnification image was processed by a slightly modified procedure of a previous report.^43^ Please refer to the Supporting Information for details of the image processing. The spots on top of the AuNT in the inset of Figure 2a might be due to the diffusion of RB photoinitiator or because of imaging variations. We observe a positive correlation between the incident laser power and the apparent quantity of BSA hydrogel immobilized over the AuNTs.

Further, regioselective immobilization is observed for lower laser power fabrication depending on the polarization of the incident laser. Figure 2a shows localization experiments where the constant incident laser electric field vector was aligned along the triangle height during the fabrication process. This results in a dipole mode with a hot spot present at the triangle tip overlapping with the polarization direction, as shown by the FDTD simulation in Figure 2d. Similarly, when the electric field vector was directed along the edge of the triangle, a dipole mode originated with hot spots present along the two tips of the triangle positioned along the field vector (Figure 2e). This association of hot spot and polarization was reflected on the regioselective positioning of BSA hydrogel at only one and two tips, respectively (Figure 2a,b). The BSA hydrogel can be observed in the SEM images as clouding over the AuNTs.^43^ The grain‐like morphology on the substrate is the result of a thin layer of Au—Pt sputtered on the nonconductive glass substrate for SEM imaging. In addition, as expected, the volumes appear similar in both cases. The regioselectivity confirms that the mechanism for BSA hydrogel immobilization is dependent on the LSPR of the nanoparticle. On doubling the incident power, we observe the coverage of all three tips of the AuNT since the minute enhancements at the misaligned tips (Figure 2e) can also result in intensities that are high enough for polymerization to occur.

Since the BSA hydrogel immobilized over the AuNTs is confined to a hot spot with dimensions well below 100 nm, direct ensemble spectroscopic measurements do not provide any inferable data. Previous reports related to ensemble measurements showed multiple broad peaks resulting from size heterogeneity and morphology changes.^30^ Also, due to the large separation between AuNTs, the signal is not high enough to be deciphered via conventional spectroscopic techniques. Hence, single particle analysis was selected as a strong tool for probing minute changes occurring over the nanoparticles.

**Figure 3:**
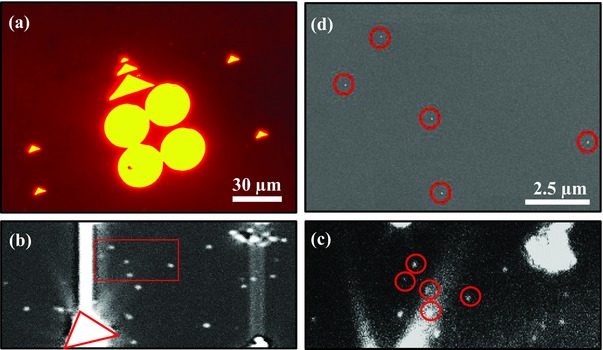
Correlated single‐particle dark‐field spectroscopy and SEM. a) Typical markers of BSA hydrogel prepared by direct laser writing to localize the individual AuNTs. b) Dark‐field image of multiple nanoparticles in the vicinity of the marker before fabrication. The extremely bright spot is the strong scattering from the marker. c) Dark‐field image of the same region after fabrication. d) SEM imaging of the region indicating the presence of well‐separated single nanoparticles.

**Figure** **3** shows the strategy employed for monitoring changes on a single particle. A Nikon TiE inverted microscope coupled to a Newton EMCCD spectroscopy detector (Andor Technology) is employed for dark‐field scattering imaging and analysis. Direct‐laser writing of the BSA hydrogel was performed using mask‐direct MPL to create unique markers for monitoring the particles during fabrication, optical characterization, and SEM. A higher incident laser power of 14 mW at the back aperture was used for direct‐laser writing of the marker. Figure 3a shows the fluorescence image (using TRITC filter) of typical BSA markers, with the desired shapes drawn and replicated on the substrate using a digital micromirror device as a dynamic mask to guide fabrication. After immobilization of AuNT on the silanized cover slip, the dark‐field image as well as spectra of 5–6 pristine AuNT particles at a region in the vicinity of the marker, were obtained (Figure 3b). MPPL was performed by scanning the laser beam over predetermined regions on the substrates where the dark‐field spectra were obtained. The fabrication process is rapid, as a 40 × 40 μm^2^ area was scanned in 5 s. Numerous layers were scanned at 0.5 μm increments to ensure the focal volume of the laser transected the AuNTs. To characterize the changes, the predetermined nanoparticles were located using the markers and the corresponding scattering spectra were obtained. Figure 3c shows the postfabrication dark‐field scattering image of the same region as in Figure 3b. The incident laser power in this case was 2 mW at the back aperture. The variation between these images is due to loosely bound particles being washed away during the fabrication and rinsing process. In order to ensure analysis of a single particle in dark‐field microscopy, the interparticle distance was tuned to be at least 1 μm, which was confirmed via correlated SEM imaging (Figure 3d).

**Figure 4:**
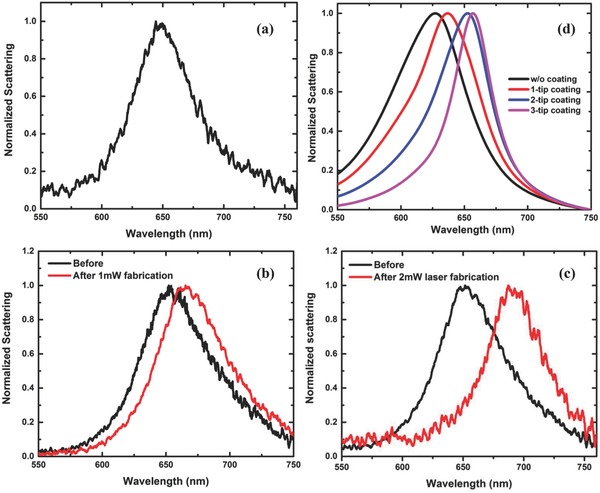
Representative dark‐field scattering spectra of a) pristine AuNT, single AuNT before and after fabrication with an incident laser power of b) 1 mW and c) 2 mW indicating a red shift post fabrication. d) FDTD simulated spectra of pristine AuNT and AuNT coated with BSA hydrogel on one, two, and three tips, respectively. Simulations were performed for AuNT of 60 nm edge length and 25 nm thickness. The BSA hydrogel with a refractive index of 1.5 was added at one, two, and three tips to mimic the experimental structures as seen in Figure 2a–c.

LSPRs are extremely sensitive to refractive index (RI) changes occurring over the nanoparticle surroundings.^51^ **Figure** **4**a shows a representative dark‐field scattering spectrum of a pristine AuNT immobilized on a glass coverslip. The spectra of four pure AuNTs were taken as a reference. Figure S2 (Supporting Information) shows the correlated dark‐field and SEM images of pristine AuNTs. The dark‐field scattering spectra of particles 1–4 are shown along with the high‐magnification SEM images in Figure S3 (Supporting Information). The peak wavelength (*λ*_max_) and full width at half maximum (FWHM) of pristine AuNTs calculated using Lorentzian peak fitting were 649 ± 2 and 63 ± 3 nm, respectively. The standard deviation observed in the peak wavelength is attributed to the variation in the size and orientation of individual particles resulting in slightly different spectra. The fabrication process was characterized using the approach described in Figure 3.

Figure 4b denotes the representative spectra of a single AuNT before and after fabrication of AuNT‐BSA hydrogel with an incident laser power of 1 mW, with spectra of four particles obtained and analyzed. A red shift of 19 ± 5 nm in *λ*_max_ was observed after immobilization of BSA hydrogel. The red shift is a consequence of the increase in the refractive index around the AuNT. The standard deviation of the peak position is significant since at such low powers, the immobilization is sensitive to the orientation of the AuNT with respect to the incident laser polarization, as seen in Figure 2a,b. Figure 4c shows representative spectra for an incident laser power of 2 mW, which results in coverage of all three tips of the AuNT with protein and a *λ*_max_ shift of 34 ± 3 nm. Since the plasmon‐induced field is localized at the tip, RI changes at distances above 15 nm do not have a strong influence on the LSPR. Thus, the effect of AuNT orientation is weaker due to the higher amount of immobilized protein. This minimizes the standard deviation observed in the case of higher laser power incidence. Our FDTD simulations support the experimentally observed resonance peak red shift upon increasing the incident light power. The simulation depicts the changes occurring in the spectra when BSA hydrogel is immobilized on one, two, and three tips, respectively (Figure 4d). The *λ*_max_ of pristine AuNT is observed at 626 nm, and a maximum shift of 30 nm is seen for BSA immobilized at all three tips. The deviation of *λ*_max_ can be due to the differences in the morphology (surface roughness, triangle shape, etc.) and medium refractive index in actual experiments and of those in the simulation.

**Figure 5:**
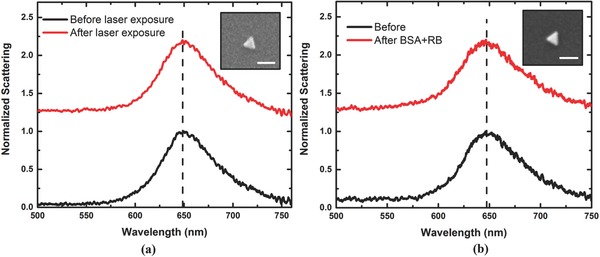
Dark‐field scattering spectra of two independent controls: a) Before and after 2 mW laser irradiation without the presence of BSA and RB and b) before and after exposure of AuNT to BSA and RB fabrication solution without any laser irradiation. The “after” plot was offset along the *y*‐axis for clarity. The inset shows the corresponding high‐magnification SEM image, with a scale bar of 100 nm.

Two independent sets of control experiments were investigated, and the results are summarized in **Figure** **5**. The first control comprises elimination of the morphology changes occurring due to laser irradiation. Figure 5a represents the spectra obtained for a single AuNT before and after irradiation using an average laser power of 2 mW, the highest power used for MPPL. The postfabrication spectra have been offset along the *y*‐axis for better clarity. No significant shift in the spectrum peak wavelength or particle morphology was observed as a result of laser irradiation. In addition, to rule out the possibility of direct adsorption of BSA or RB molecules on AuNTs, the spectral response of AuNT was tracked before and after immersion in BSA fabrication medium. Techniques such as LAMI are less effective since it relies on effective coverage of proteins prior to fabrication.^43^ In contrast, due to the low fabrication times involved in MPPL, we observed no shift in the AuNT peak position and no morphology changes (Figure 5b).

**Figure 6:**
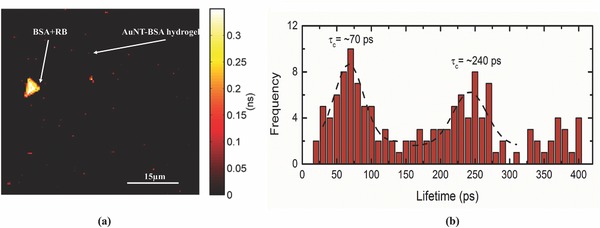
FLIM of the AuNT‐BSA hydrogel nanoparticles fabricated using 2 mW incident laser. a) FLIM image showing the control BSA hydrogel and AuNT‐BSA hydrogel in the same FOV. b) Histogram of lifetimes obtained from the same FOV indicating two separate lifetimes of ≈70 and ≈240 ps for AuNT‐BSA hydrogel and control BSA hydrogel, respectively.

To further confirm AuNT‐BSA hydrogel fabrication, multiphoton FLIM was employed. FLIM is built upon time‐resolved detection and can provide a sensitive contrast mechanism to identify the local environment of a fluorophore.^52^ Using FLIM, it was determined that two distinct lifetime populations are present for AuNT‐BSA hydrogel and BSA hydrogel. With the aid of markers, the region of AuNT‐BSA hydrogel fabricated with 2 mW was analyzed using FLIM (refer to Figure 2c for the dark‐field scattering image). The observed two‐photon fluorescence signal is primarily due to the RB photosensitizer. Although there have been various reports of a strong influence of the surfactant molecules present over Au nanoparticles on the lifetime of RB,^53^ this effect is negligible in the present case since in addition to regular rinsing of the substrate, the AuNT solution was centrifuged and redispersed in water twice to remove CTAC prior to immobilization on the glass coverslip. **Figure** **6** shows the FLIM image along with a histogram of lifetime distribution within the field of view, which contains the BSA hydrogel fabricated using MPL and the AuNT‐BSA complexes fabricated by MPPL. A decrease in the fluorescence lifetime is observed over the particles, as compared to the control structure. The lifetime histogram reveals an average lifetime of ≈70 ps for AuNT‐BSA hydrogel particles, whereas the control BSA hydrogel has an average lifetime of ≈240 ps. The presence of two lifetime populations at ≈240 and ≈370 ps for control BSA hydrogel is likely due to self‐quenching of RB molecules.^54^ The reduction in lifetime may be attributed to the resonance energy transfer from the fluorophore to the AuNT, confirming the immobilization of BSA hydrogel on the AuNT.^53^, ^55^

## 3 Conclusions

In conclusion, we employed MPPL to rapidly localize BSA hydrogel molecules at the tips of AuNTs. We further demonstrated regioselective positioning along with control over the amount of protein deposited on the AuNTs. A strong dependence on the LSPR is established resulting in polarization‐dependent positioning of the molecules over AuNTs, as is evident from SEM images. We also tracked the changes occurring on a single AuNT using dark‐field scattering spectroscopy and compared the experimental results with FDTD simulations. A red shift in the plasmon resonance peak wavelength, which is correlated with the incident laser power, confirms the immobilization of BSA hydrogel over AuNT. FLIM imaging was performed for additional confirmation of the immobilization. Our rapid and single‐step protein immobilization technique along with single‐nanoparticle dark‐field monitoring can act as a framework to better understand light–molecule interactions at the subnanoparticle level. These precisely controllable nanoparticle–biomolecule complexes have potential applications in nanophotonics, nanomedicine, and life sciences.

## 4 Experimental Section

*Chemicals*: CTAC (25 wt% in water), hydrogen tetrachloroaurate trihydrate (HAuCl_4_.3H_2_O, >99.9%), sodium iodide (>99.5%), sodium borohydride (NaBH_4_, 99%), 3‐(mercpatopropyl)triethoxysilane (MPTES, 95%), Rose Bengal (RB, 33000), RBS 35 detergent, and ethanol (>99.9%, absolute grade) were purchased from Aldrich. Toluene (>99.5%), hydrochloric acid (37%), acetone (>99.9%), methanol (>99.8%), and microscopic cover glass (12‐545‐B, size #1) were procured from Fisher Scientific. BSA (BAH64) was obtained from Equitech‐Bio. All chemicals were used without further purification. Deionized water was used during all experiments.

*Synthesis of Gold Nanotriangles*: The AuNTs were prepared following the procedure of Scarabelli et al.^49^ Briefly, Au seed@CTAC were prepared adding, under vigorous stirring, 300 μL of a 0.01 m NaBH_4_ solution to a 4.7 mL solution 0.1 m in CTAC and 0.25 × 10^−3^ m in HAuCl_4_. The prepared seed were aged for 2 h at room temperature and then diluted ten times in CTAC 0.1 m before proceeding. 10 mL of AuNT solution was prepared as follows. Two growth solutions were simultaneously prepared, the first (1) made of 8 mL of Milli‐Q water, 1.6 mL of a 0.01 m CTAC solution, and 40 μL of a 50 × 10^−3^ m HAuCl_4_ solution, and the second (2) made of 10 mL of 0.05 m CTAC, 125 μL of a 50 × 10^−3^ m HAuCl_4_ solution, and 75 μL of a 0.01 m NaI solution. 40 and 100 μL of a 0.1 m ascorbic acid solution were added to solution (1) and (2), respectively, and manually mixed until complete disappearance of the yellow color (few seconds). Immediately after reaching complete transparency, 100 μL of the diluted seed@CTAC was added to solution (1). After manually stirring the solution for 1 or 2 s, 1.2 mL of solution (1) was injected into solution (2) (and manually stirred for few seconds). The growing particles were then left set undisturbed for at least 1 h before characterization. The shape‐yield in AuNT was about 60%. In order to separate the byproduct, 2.75 mL of a concentrated CTAC solution (25% in weight, ≈0.78 m) was added to the AuNT solution; the entire mixture was transferred in a cylinder and left undisturbed overnight. Interparticle depletion forces drove the flocculation and precipitation of the AuNTs. The supernatant (pink‐purple) was discarded, and the precipitate (forming a black patina at the bottom of the cylinder) was redispersed with 4 mL of CTAC 0.05 m, immediately giving rise to a deep blue coloration. The produced AuNTs presented a plasmonic band centered at 633 nm, an edge length of 57 ± 4 nm, and a thickness of 27 ± 2 nm.

*Glass‐MPTES‐AuNT Preparation*: A slightly modified procedure of a previous report was used.^56^ Briefly, before the MPTES functionalization, size #1 glass coverslips were cleaned with RBS detergent at 90 °C for 30 min. Then, the slides were immersed in bidistilled water and sonicated for 5 min followed by thorough rinsing. The coverslip was dried under N_2_ flow and immersed in 1:1 v/v methanol and HCl solution for 25 min. The washed slides were dried in an oven (140 °C) for 1 h and then allowed to cool to room temperature in air. Then, the slides were fully immersed in a 5% (v/v) solution of MPTES in toluene and allowed to react for 4 h at 40 °C. After 4 h, toluene solution was removed and a three‐cycle washing procedure was carried out by sonicating for 3 min in a beaker with (i) pure toluene, (ii) 1:1 v/v toluene/ethanol, and (iii) pure ethanol. Finally, the slide was dried under N_2_ flow. AuNT solution was centrifuged (4500 rpm for 10 min) and redispersed twice in water. The solution was casted onto a freshly prepared silanized coverslip for 2 h to obtain well‐separated AuNTs on silanized glass. The functionalized coverslips were used within 1 h for fabrication.

*AuNT‐BSA Hydrogel Fabrication*: Fabrication was performed on the AuNTs immobilized on coverslips using a previously reported setup.^57^ The output of a mode‐locked titanium:sapphire laser (Coherent, Mira 900F) operating at 740 nm was scanned in two axes using a galvanometer driven scan mirror (Leica, TCS‐4D). Following the scan mirror, lenses collected the light and focused the laser onto a digital micromirror device (BenQ, MP510) consisting of an array of individually addressable, aluminum mirrors that can be switched between “on” and “off” states using a digital input from a computer (binary image). The light reflected from mirrors in the “on” state travels further down the optical path, and into an inverted microscope outfitted with a Zeiss Fluar, 100×/1.3 NA, oil immersion objective. The laser power was attenuated with a half‐wave plate/polarizing beam splitting cube pair to achieve an average power of 1–2 mW, measured at the back aperture of the objective. Beginning slightly below the coverslip, the laser was raster scanned over an ≈(40 × 40) μm^2^ area for fabrication. A single plane was scanned in 5 s, and up to seven additional focal planes (axially spaced 0.5 μm apart and further stepped into fabrication solution) were scanned using a motorized stage to ensure AuNTs were exposed to the focal volume of the laser. Fabrication solution consisted of BSA at 400 mg mL^−1^ and 1 × 10^−2^ m Rose Bengal as a photosensitizer. In order to monitor single particles, unique fluorescent markers of BSA hydrogels (Figure 2a) were initially fabricated on silanized glass coverslips using direct laser writing with an average laser power of 14 mW at the back aperture of the microscope.

*Dark‐Field Scattering Microscopy*: A Nikon TiE inverted microscope coupled to a Shamrock 303 imaging spectrograph, which contains a Newton EMCCD spectroscopy detector (Andor Technology Plc.), was utilized to study the single‐nanoparticle scattering spectrum of AuNTs and protein modified AuNTs. An oil immersion dark‐field condenser (Nikon) and a 100× oil immersion objective (CFI Plan Fluor, Nikon) were utilized to capture the image of the sample. A halogen lamp and Xe lamp (Nikon) were used for performing dark‐field scattering and fluorescence imaging, respectively. The fluorescence imaging was used only to locate the unique markers fabricated using direct laser writing. Careful sample preparation ensured that the particles were well separated. Commercial software, Solis (Andor), was utilized to control the spectrograph, spectroscopy detector, and microscope to capture the signal of a single AuNT and record the single‐nanoparticle scattering spectrum.

*Fluorescence Lifetime Imaging Measurements*: FLIM was performed on the sample based on time‐correlated single‐photon counting (TCSPC) technique. The complete setup details can be found in a previous report.^58^ Key components pertaining to lifetime measurements include the femtosecond titanium:sapphire laser tuned to 800 nm (≈200 fs) (Mira 900, Coherent), galvo scanning mirrors (6215H, Cambridge Tech.), and a GaAsP photomultiplier tube (PMT) (H7422PA‐40, Hamamatsu) in non‐descanned detection scheme. The output current of the PMT is amplified using a preamplifier (HFAC‐26, Becker and Hickl GmbH) prior to reaching the photon counting board (SPC‐150, Becker and Hickl GmbH). Data were acquired in custom LabView interface, while processing and visualization of the FLIM datasets were performed in Matlab. Fluorescence lifetimes were recorded with a 20 ps time resolution and integration time of 5 ms using an average laser power of 2 mW. Lifetimes were fit using a least squares model of a single exponential decay.^59^ Data points with fewer than 200 photons were removed from the fitting, and fits with *χ* ^2^ values less than 1.5 were discarded to ensure fit quality.

*Scanning Electron Microscopy*: After all the above characterizations, SEM (FEI Quanta 650) images of the samples were obtained to validate the single‐particle measurements. Samples were sputter coated with a thin Au—Pt layer prior to imaging.

*FDTD Simulations*: Numerical simulations for the pristine AuNT and AuNT‐BSA hydrogel were performed using commercially available package FDTD Solutions from Lumerical Inc. AuNT of edge length 60 nm and thickness of 25 nm (Figure S1b, Supporting Information) was used. To mimic the unpolarized light used in experiments, circularly polarized light with normal incidence as the light sources was used. 2D detector was used to calculate the scattering spectra. Perfectly matched layers (PMLs) were applied at all boundaries. The RI of the glass coverslip was set as 1.52 and the optical constants of gold and BSA hydrogel (RI = 1.5) were adapted from the literature.^60^, ^61^ The BSA hydrogels were added at one, two, and three tips to characterize the changes in the spectra.

## Supporting information

As a service to our authors and readers, this journal provides supporting information supplied by the authors. Such materials are peer reviewed and may be re‐organized for online delivery, but are not copy‐edited or typeset. Technical support issues arising from supporting information (other than missing files) should be addressed to the authors.

Supplementary
