# Supplementary material for: Regioselective Localization and Tracking of Biomolecules on Single Gold Nanoparticles
Source: Adv Sci (Weinh). 2015 Sep 28;2(11):1500232. doi: 10.1002/advs.201500232 (PMC5019259; doi:10.1002/advs.201500232)
Supplement: Supplementary file 1 — Supplementary [file ADVS-2-0q-s001.pdf]

Copyright WILEY-VCH Verlag GmbH & Co. KGaA, 69469 Weinheim, Germany, 2015.

ADVANCED SCIENCE
Open Access

Supporting Information

for *Adv. Sci.*, DOI: 10.1002/advs.201500232

Regioselective Localization and Tracking of Biomolecules on Single Gold Nanoparticles

*Bharath Bangalore Rajeeva, Derek S. Hernandez, Mingsong Wang, Evan Perillo, Linhan Lin, Leonardo Scarabelli, Bharadwaj Pingali, Luis M. Liz-Marzán, Andrew K. Dunn, Jason B. Shear, and Yuebing Zheng**

WILEY-VCH

Copyright WILEY-VCH Verlag GmbH & Co. KGaA, 69469 Weinheim, Germany, 2013.

Supporting Information

Regioselective Localization and Tracking of Biomolecules on Single Gold Nanoparticles

*Bharath Bangalore Rajeeva, Derek S. Hernandez, Mingsong Wang, Evan Perillo, Linhan Lin, Leonardo Scarabelli, Bharadwaj Pingali, Luis M. Liz-Marzán, Andrew K. Dunn, Jason B. Shear, and Yuebing Zheng**

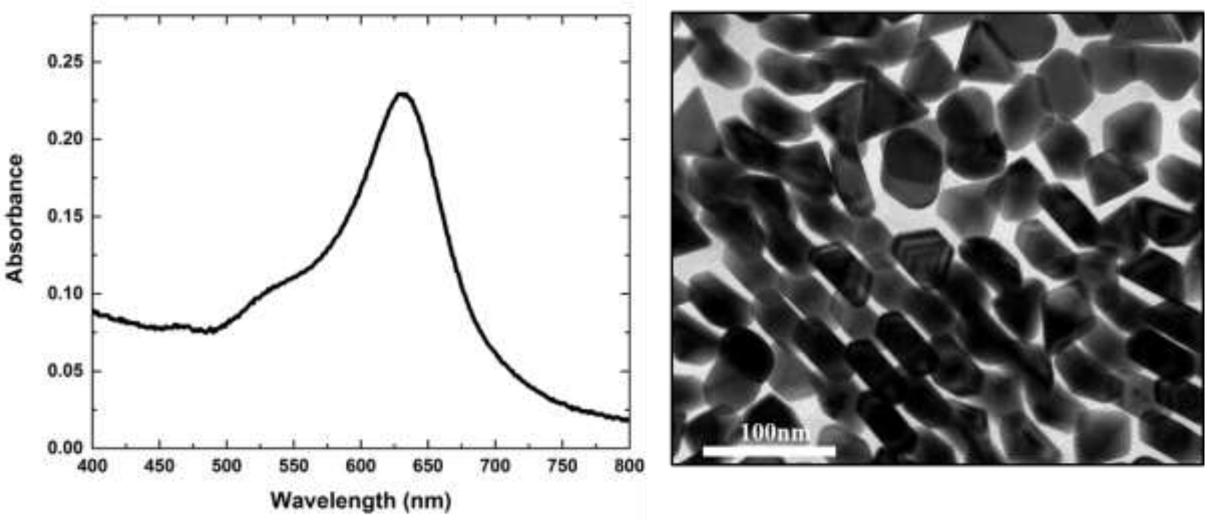

Figure S1: (a) The absorbance spectra of colloidal AuNT solution measured using Ocean optics UV-Vis-NIR spectrometer. (b) Transmission electron microscopic (TEM) image of the AuNTs showing thickness of 25±2nm.

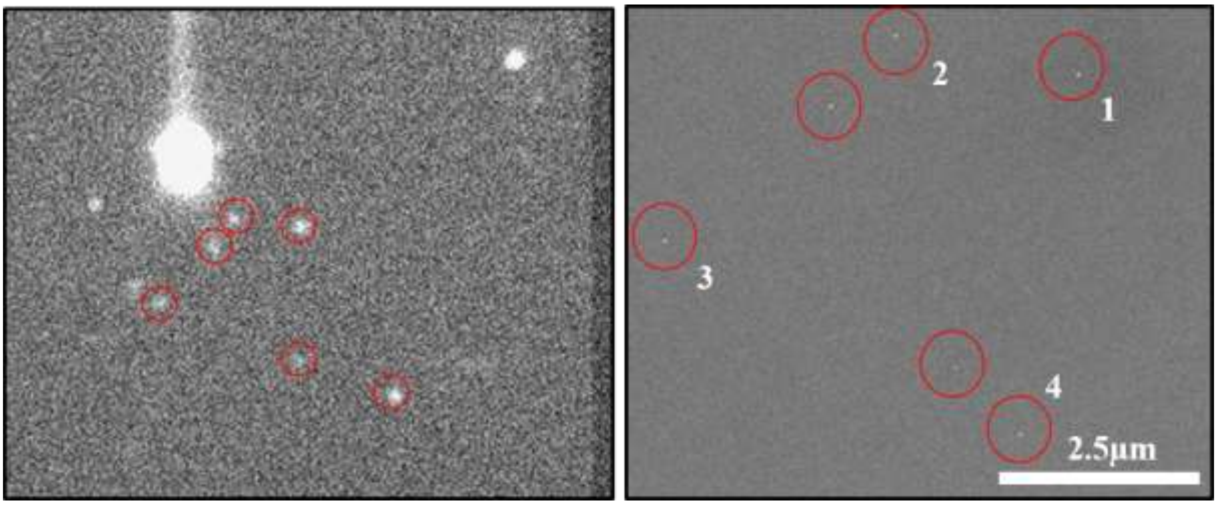

Figure S2: Correlated (a) dark-field scattering image and (b) Correlated SEM image of pristine AuNT showing adequate separation between particles.

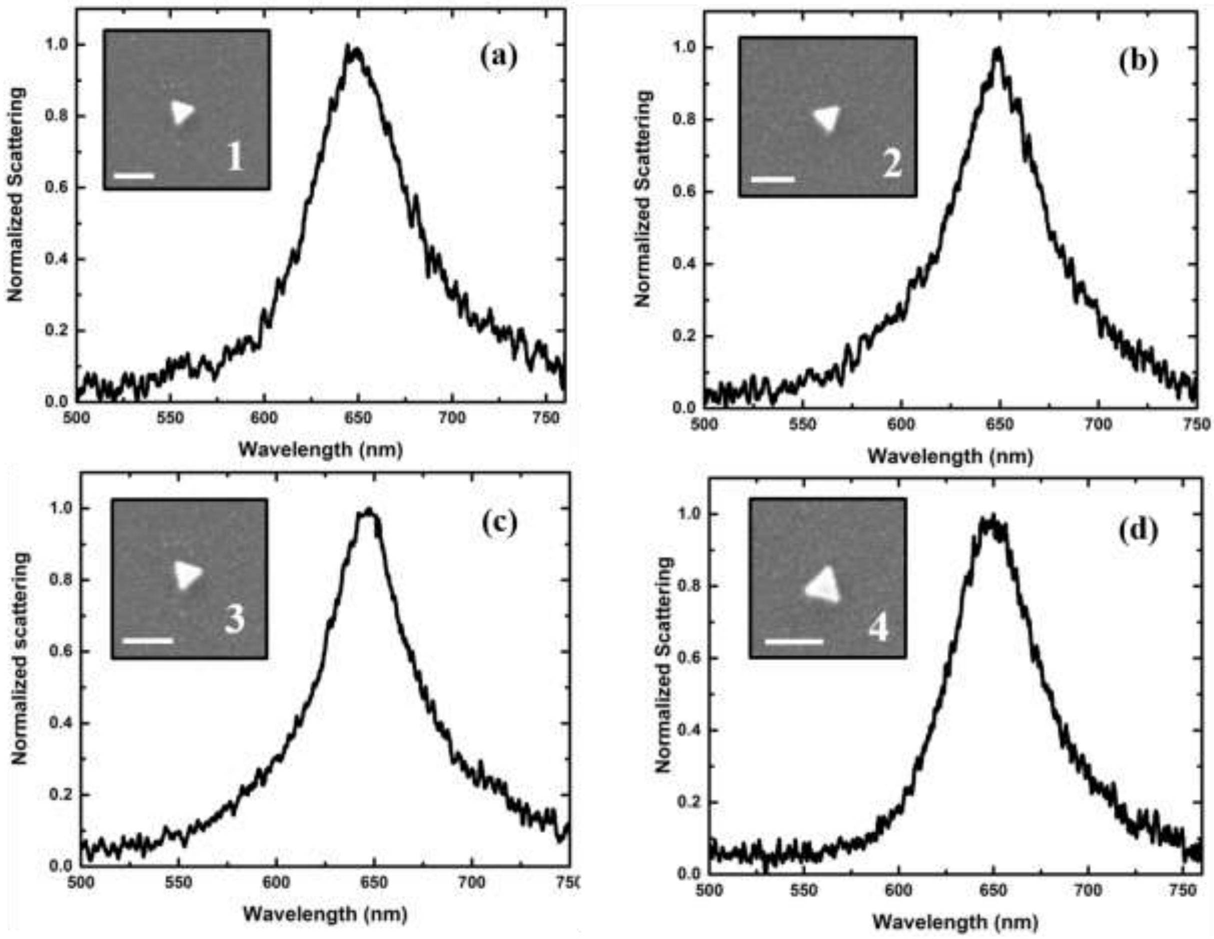

Figure S3: The normalized dark-field scattering spectra of particles (a) 1 (b) 2 (c) 3 and (d) 4 labelled in supplementary figure 2. The inset of each plot has the high magnification SEM image of the pristine AuNT. The scale bar is 100nm. The peak position of the spectra calculated using Lorenzian fit is 649±2.2nm.

The close proximity of BSA hydrogels to the AuNT mandates further processing of the image to clearly locate the position of BSA hydrogels. Figure S4 demonstrates the procedure employed for processing Figure 2(c). First, the AuNT-BSA hydrogel complex was selected and overlaid on a white background. Next, the histogram of the gray level (GL) of the image was obtained. As expected, due to the white background, majority of GL count is observed at 255. A magnified histogram is shown in Figure S4(b). Since the brightest component is expected to be the AuNT particle, a threshold was set to separate the AuNT from the BSA hydrogel. Figure S4(c,d) show the isolated BSA location before and after adding color. A similar procedure is repeated for the other scenarios.

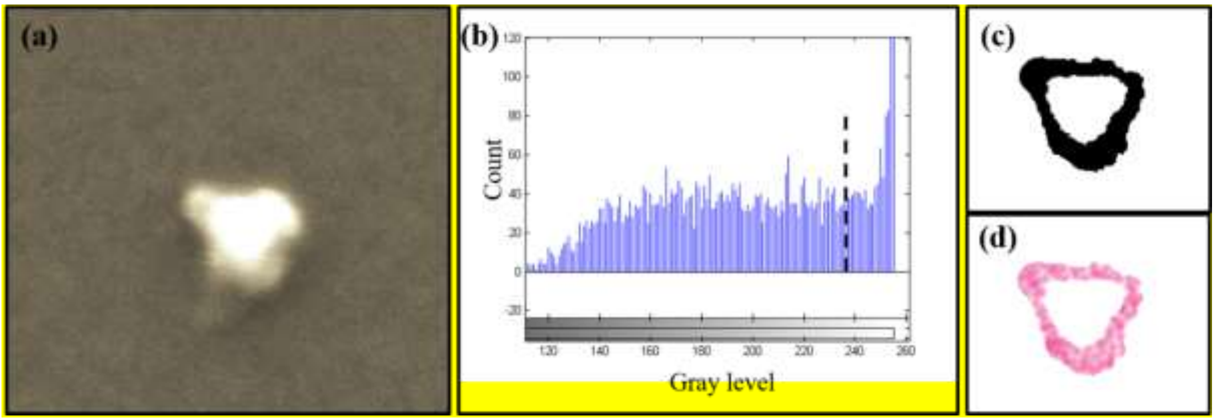

Figure S4: The stages of image processing (a) Original SEM image depicting coverage of BSA at all three tips. (b) Histogram of the processed image of AuNT-BSA hydrogel overlaid on a white background. The black line depicts the threshold value set to differentiate BSA and AuNT. (c) Processed image depicting only the pixels pertaining to BSA (d) A colored representation of the same region.
